# Shared and organ-specific gene-expression programs during the development of the cochlea and the superior olivary complex

**DOI:** 10.1080/15476286.2023.2247628

**Published:** 2023-08-21

**Authors:** Mor Bordeynik-Cohen, Michal Sperber, Lena Ebbers, Naama Messika-Gold, Constanze Krohs, Tal Koffler-Brill, Yael Noy, Ran Elkon, Hans Gerd Nothwang, Karen B. Avraham

**Affiliations:** a Laboratory of Neural and Sensory Genomics, Department of Human Molecular Genetics & Biochemistry, Faculty of Medicine and Sagol School of Neuroscience, Tel Aviv University, Tel Aviv, Israel; b Department of Human Molecular Genetics & Biochemistry, Faculty of Medicine, Tel Aviv University, Tel Aviv, Israel; c Neurogenetics group and Cluster of Excellence Hearing4All, School of Medicine and Health Sciences and Research Center for Neurosensory Science, Carl von Ossietzky University Oldenburg, Oldenburg, Germany

**Keywords:** MicroRNAs, transcriptomics, gene regulatory networks, inner ear, brainstem

## Abstract

The peripheral and central auditory subsystems together form a complex sensory network that allows an organism to hear. The genetic programs of the two subsystems must therefore be tightly coordinated during development. Yet, their interactions and common expression pathways have never been systematically explored. MicroRNAs (miRNAs) are short non-coding RNAs that regulate gene expression and are essential for normal development of the auditory system. We performed mRNA and small-RNA sequencing of organs from both auditory subsystems at three critical developmental timepoints (E16, P0, P16) to obtain a comprehensive and unbiased insight of their expression profiles. Our analysis reveals common and organ-specific expression patterns for differentially regulated mRNAs and miRNAs, which could be clustered with a particular selection of functions such as inner ear development, Wnt signalling, K+ transport, and axon guidance, based on gene ontology. Bioinformatics detected enrichment of predicted targets of specific miRNAs in the clusters and predicted regulatory interactions by monitoring opposite trends of expression of miRNAs and their targets. This approach identified six miRNAs as strong regulatory candidates for both subsystems. Among them was miR-96, an established critical factor for proper development in both subsystems, demonstrating the strength of our approach. We suggest that other miRNAs identified by this analysis are also common effectors of proper hearing acquirement. This first combined comprehensive analysis of the developmental program of the peripheral and central auditory systems provides important data and bioinformatics insights into the shared genetic program of the two sensory subsystems and their regulation by miRNAs.

## Introduction

The ability to perceive sound is the result of a complex sensorineural processing that spreads from the inner ear to the central nervous system. Hearing loss is the most prevalent sensory deficiency in humans [1,2], and affects 1.5 billion people [3]. Although about 50% of deafness cases are estimated to be genetic-based [4], in many cases the cause remains unresolved [5,6]. A deeper understanding of the precise orchestrated genetic program governing hearing is vital for optimal genetic screening and development of genetic therapeutics [7].

**Figure 1.**
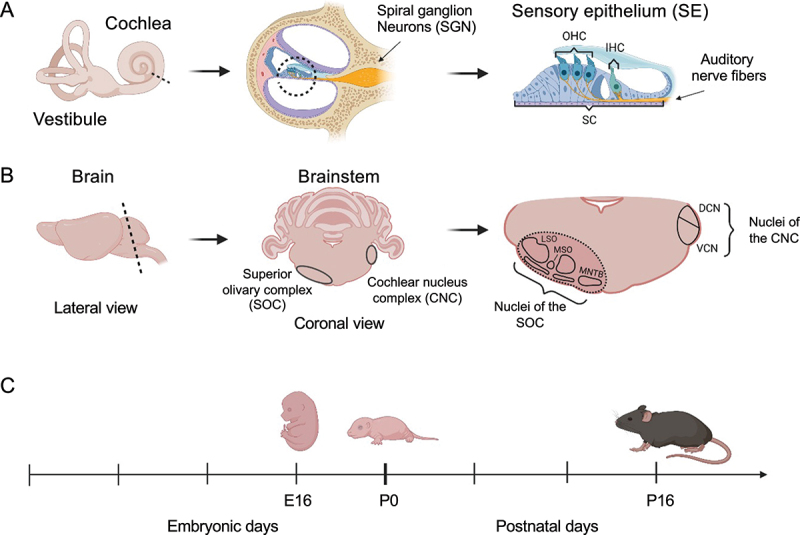
Illustration of the mice organs dissected for RNA analysis. (a) The inner ear, containing the sensory epithelium (SE). OHC – outer hair cells, IHC – inner hair cells, SC – supporting cells. (b) Central auditory structures on the level of the brainstem include the CNC and SOC. Harvested brain area marked with dashed line. SOC and CNC are present on either side of the brain. MSO – Medial superior olive, LSO – Lateral superior olive, MNTB – Medial nucleus of the trapezoid body, DCN – dorsal cochlear nucleus, VCN – ventral cochlear nucleus. Created with BioRender.com. Images not drawn to scale relative to one another. (c) Timeline of dissected organs.

The auditory system is composed of the peripheral and the central auditory systems. The former receives sound waves and transforms them into mechanical movements and subsequently into electrical signals in the organ of Corti, the sensory epithelium (SE), of the inner ear (Figure 1). In contrast, the central auditory system is implicated in the processing and perception of auditory stimuli. Signals from the inner ear are relayed through the spiral ganglion neurons forming the auditory nerve to the brainstem. Upon entering the brainstem, the auditory nerve fibres bifurcate innervating different subdivisions of the cochlear nucleus complex (CNC), which represents the first station of central sound processing. The posterior branch reaches into the area of the dorsal cochlear nucleus (DCN), whereas the anterior branch innervates the ventral cochlear nucleus (VCN). From here the auditory information is further distributed within the central auditory system [8]. Neurons from the VCN primarily project to the superior olivary complex (SOC), which is important for processing fundamental features of the sound stimulus including sound duration and localization [9,10]. The SOC consists of three primary nuclei, the lateral and medial superior olive (LSO and MSO, respectively) and the medial nucleus of the trapezoid body (MNTB), as well as three periolivary nuclei (Figure 1).

Beyond the brainstem, the signal is further processed in the inferior colliculus in the midbrain and the medial geniculate body of the thalamus before reaching the primary auditory cortex, where final processing and perception of the sound takes place. In addition to the ascending auditory pathway, the SOC also harbours a top-down system, the olivocochlear system, which sends feedback from the brainstem to the inner ear [11]. Because of the tight functional interplay between the peripheral and central auditory system it has been suggested that their development is governed by a shared genetic program [12]. For example, the inner ear and the auditory nerve derive both from the otic placode, i.e. the same developmental anlage [13,14]. Accordingly, a temporal gene expression analysis of the SOC has revealed enrichment of known deafness-associated genes [15], and 24 genes have currently been shown to play an intrinsic role in both systems [16].

One such shared genetic factor is the microRNA (miRNA)-96 (miR-96), where point mutations in this miRNA give rise to irregular hair bundles in the inner ear and result in peripheral deafness in both humans and mice [17,18]. These peripheral alterations are accompanied by dramatic changes in the auditory brainstem, including a decrease in the volume of SOC nuclei and impaired maturation of the calyx of Held, a large axosomatic auditory synapse [19]. miRNA expression profiles exhibit high cell-type specificity and are crucial for nervous system and developmental cell diversity [20]. In particular, miRNAs are critical for the embryonic formation of the cochlea [21] and the SOC [22]. In total, more than 400 miRNAs are known to be expressed in the mouse inner ear [23] and abundant cochlear miRNAs are also expressed in the SOC [24].

We have now employed RNA- and small RNA sequencing at three developmental stages of the inner ear SE and the SOC in order to conduct a systematic investigation of common and organ-specific gene expression programs of the peripheral and central hearing systems. Our integrated transcriptomic analysis was able to delineate the main gene regulatory networks that drive cochlea and SOC development, establish putative regulatory interactions between miRNAs and their target genes in these processes, and identify six miRNAs as key regulators of developmental programs in both systems. Further study of these miRNAs holds the potential to uncover new expression regulation pathways that are essential for both the peripheral and central hearing systems.

## Materials and methods

### RNA isolation

All procedures involving animals were performed in accordance with the regulations of German Federal Law concerning the care and use of laboratory animals, and the guidelines of the EU Directive 2010/63/EU for animal experiments or guidelines set forth by the National Institutes of Health Guide for the Care and Use of Laboratory Animals. Protocols were approved by the local animal care and use committee (LAVES, Oldenburg) or the Animal Care and Use Committee at Tel Aviv University (01-17-098). Cochlear SE were dissected from 12 inner ears of 6 C57BL/6JOlaHsd mice (Envigo, Jerusalem, Israel) of both sexes at E16, P0, and P16. Total RNA was isolated, using the Direct-zolTM RNA Miniprep kit (Zymo Research, CA, USA). SOC tissue was obtained by decapitation of C57Bl6/J mice of both sexes at P0, or at P16 from animals that were sacrificed with CO2 and subsequently decapitated. In both cases, the brain was immediately dissected out of the skull and frozen on dry ice. Alternatively, a C-section was performed on anesthetized time-pregnant mice, and E16 embryos were decapitated, and the heads were frozen on dry ice. The SOC region was cut out on a cryostat and the tissue of several animals was collected for RNA isolation. Eight E16 embryos were used per sample, with 6–8 animals used for P0 samples, and 3 animals for each P16 sample. RNA was isolated with the innuPrep miRNA Kit (Isolation of miRNAs and total RNA) and frozen at −80°C. RNA was collected in two to four biological repeats for every developmental stage.

### RNA sequencing

Cochlear RNA libraries were prepared and sequenced at the Genomics Research Unit supported by the Alfredo Federico Strauss Center for Computational Neuro-Imaging at Tel Aviv University. The NEBNext® Ultra II Directional RNA Library Prep Kit (Illumina) with NEBNext rRNA Depletion Kit v2 was used for library preparation and the NextSeq 500/550 High Output Kit v2.5 for RNA sequencing. Cochlear small-RNA sequencing was carried out by Macrogen using the SMARTer smRNA-Seq Kit (Takara Bio Inc.) for library preparation, and Illumina HiSeq2500 Rapid Run mode, 50 bp single-end configuration, 120 million reads per lane, for sequencing. The SOC library preparation and next-generation sequencing was done by Macrogen, Korea; 1 μg of total RNA was used for each mRNA sequencing reaction, and 3 μg of total RNA for each miRNA sequencing reaction. Each sample was prepared in biological triplicates. Technical requirements for the sequencing reactions were as follows: mRNA/: Library type: Illumina transcriptome; library: Illumina TruSeq Stranded Total RNA with Ribo-Zero Gold Human/Mouse/Rat; sequencing: NovaSeq; sequencing coverage: 60 million reads/sample; read length: 100 bp paired end. miRNA: Library type: Illumina transcriptome; library: Illumina TruSeq Small RNA library; sequencing: HiSeq2500 rapid run mode 1 × 50bp lane-based seq; sequencing coverage: 1/6 lane (120 million reads/lane = 20 million reads/sample); read length: 50 bp single end. The sequencing data was delivered as raw data FASTQ files.

### RNA-seq analysis

Alignment and read counting per gene were performed using STAR [25], the mm10 mouse reference genome, and GENCODE gene annotations. Gene read counts were converted to CPM units. Overall, 19908 genes (of which 15,409 (77%) are protein coding) were detected in our dataset with over 0.5 CPM in three replicates of any time point (in either the cochlea or the SOC). PCA and differential expression analysis were performed using DESeq2 [26]. The definition of a differentially expressed gene was based on three criteria: FDR < 1%, fold change > 2.5, and a consistency in the direction of the expression change (up or down-regulation compared to E16) over all replicates. This system detected a total of 3,717 down-regulated and 4,333 up-regulated genes in our dataset, which were clustered using CLICK (implemented in EXPANDER) [27]. Gene Ontology (GO) enrichment tests used the clusterProfiler package [28] (with the parameters: Ontology – biological process, p-value adjustment method – false discovery rate, p-value cut-off − 1%, adjusted p-value cut-off − 5%). Heatmaps were generated using Heatmapper [29].

### miR-seq analysis

Alignment was performed using Bowtie [30] and a reference we produced from all the mouse miRNAs in MirGeneDB [31]. Overall, the expression of 319 miRs were detected in our dataset, with over 1 CPM in at least 2 replicates of any time point in either the cochlea or the SOC. PCA and differential expression analysis were performed using DESeq2. Differentially expressed miRs were defined as those with a fold change > 1.5, FDR < 5%, and a consistent up or down-regulation in either P0 or P16 compared to E16 in all replicates. According to these criteria, 215 and 193 miRs were called as differentially expressed in the cochlea and in the SOC, respectively, with a total of 223 unique differentially expressed miRs. Predicted miR targets were downloaded from TargetScan [32]. A hyper-geometric test was performed on each cluster of down or up-regulated genes and every miR, in order to identify significant over-representation of predicted miR target genes in the differentially expressed gene cluster. These enrichment tests were performed for miRs with an expression pattern that was negatively correlated with the mean expression pattern of the predicted targets assigned to the cluster.

## Results

### Transcriptional gene expression shared or specific to cochlea and SOC

Our first goal was to delineate developmental gene-expression programs that are either shared by the cochlea and SOC or are specific to one of these organs. For this purpose, we used RNA-seq to profile the transcriptome of these two organs (Figure 1) at three developmental time points (E16, P0, and P16). These time points were chosen because in the SOC, migration of neurons is completed at E16 and the auditory brainstem neurons begin to functionally mature within their functional circuits at P0 [15]. In contrast, SE cells exit the cell cycle at E16 and complete final differentiation at P0. In both organs, P16 is a timepoint after hearing onset and represents the adult-like tissue [33]. Each biological condition was probed using 3–4 independent samples.

**Figure 2.**
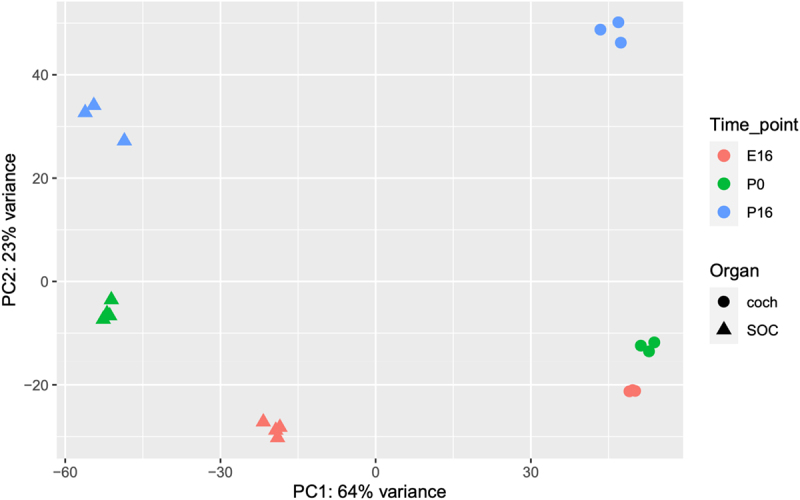
Principal component analysis (PCA) of the RNA-seq data. The main component (PC1) is associated with organ-specific expression programs, while the second component (PC2) reflects developmental programs that are shared by the cochlea and the SOC.

**Figure 3.**
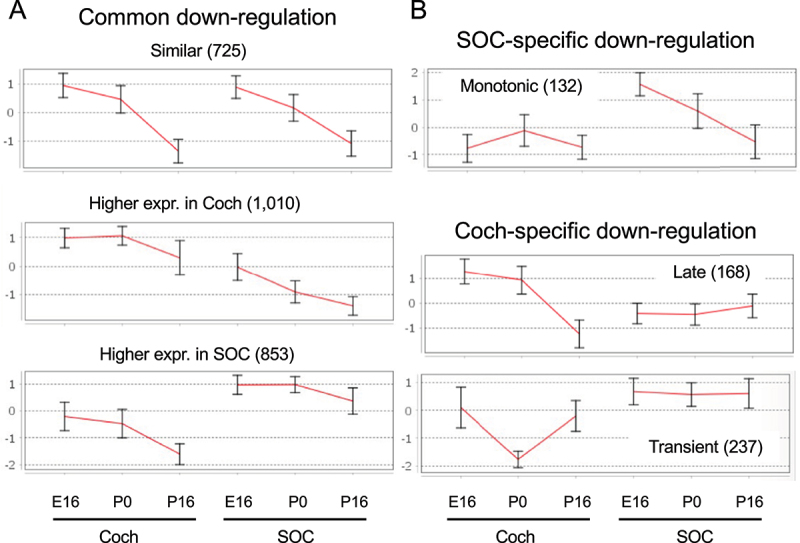
Main clusters of the DEGs repressed during development of the auditory system. (a) Clusters of genes whose expression is repressed during development in both the cochlea and the SOC (albeit with different absolute levels in the two organs). (B) Clusters of genes whose expression is repressed during development in only one of the organs. Each cluster is represented by the mean expression pattern of the genes assigned to it. Error bars represent ± SD. Gene expression levels were standardized to mean 0 and SD 1 prior to clustering.

Overall, the expression of 19,908 genes could be detected robustly (Table S1–2). PCA revealed that the organ was the main influence on gene expression profiles, with developmental time a secondary factor (Figure 2). Differential expression analysis identified 5,017 and 4,925 differentially expressed genes (DEGs; FDR = 1%) whose expression level was significantly altered during development in the cochlea and SOC, respectively (Table S3). The expression patterns of the DEGs were very varied, and cluster analysis was applied to divide them into distinct groups according to their expression profile across the probed conditions (Table S4). Focusing first on the DEGs with repressed expression during development, we detected three main clusters of DEGs whose expression declined in both organs (with either similar or different initial levels at E16, Figure 3a). Gene ontology (GO) enrichment analysis indicated that the cluster of 725 repressed DEGs with similar basal expression at E16 is significantly enriched for genes that function in all cell-cycle phases (Table 1; Table S5A). In contrast, the cluster of 1,010 DEGs with reduced expression in both organs at the third time point (P16), but with markedly higher expression levels in the cochlea, is significantly enriched for genes that function in early inner-ear development and the Wnt receptor-signalling pathway (Table 1; Table S5A). This gene cluster also reflected the general repression of differentiation programs of multiple tissues (including the skeletal system, kidney, artery, limb, skin, and lung) that takes place in the cochlea and SOC as these organs mature. On the other hand, a cluster of 853 DEGs that were similarly repressed in both organs at the last time point (P16) but with markedly higher expression levels in the SOC, is enriched for genes that play key roles in axonogenesis/axon guidance.

**Table 1.** GO enrichment for clusters of repressed DEGs.

Cluster	GO category	EF	pval	pval.adj
Common – higher expression in cochlea	inner ear development	3.8	3.41E–14	5.61E–12
	Wnt receptor signalling pathway	2.84	6.27E–14	9.52E–12
Common – higher expression in SOC	axon guidance	3.22	2.44E–08	4.89E–05
Common – similar	DNA replication	3.4	5.71E–11	6.65E–09
	meiotic cell cycle	3.55	2.00E–10	2.06E–08
	G2/M transition of mitotic cell cycle	3.75	2.37E–06	9.64E–05
Cochlea-specific – transient	sensory perception of sound	7.33	3.90E–06	6.30E–03

Three additional main clusters identified as associated with organ-specific repression programs are presented in Figure 3b. These are a cluster of 132 genes specifically expressed and monotonically repressed in the SOC, which is enriched for genes that function in muscle cell development and myotube differentiation, a cluster of 168 genes specifically expressed in the cochlea and repressed at P16 and enriched for genes that function in sensory perception of sound, and last a cluster of 237 genes transiently repressed in the cochlea and enriched for genes that function in sensory perception of sound (Table 1; Table S5A).

**Figure 4.**
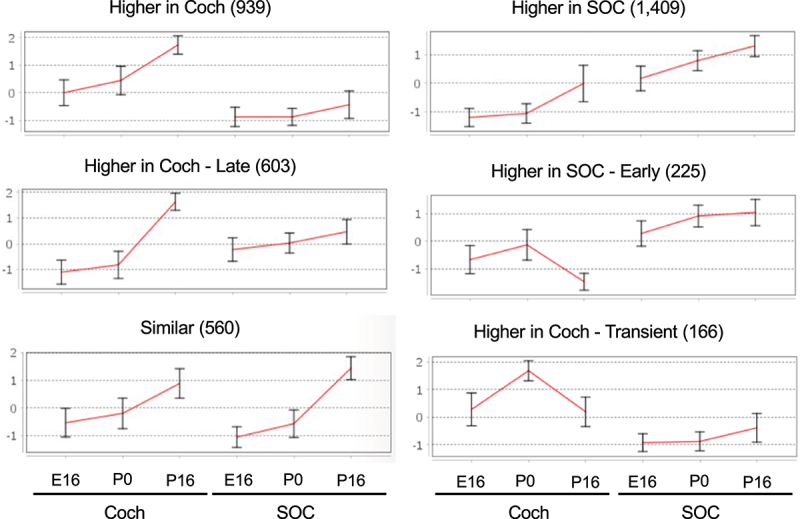
Main clusters of the DEGs induced during development of the auditory system. Same as Figure 3, but for genes whose expression level is increased during development.

Two clusters of DEGs (939 and 603 genes) that are induced during the development of the auditory system and are more prevalent in the cochlea, were identified as enriched for genes that function in sensory perception of sound (earlier induction), potassium ion transport (later induction), and T-cell mediated immune responses (Figure 4; Table 2; Table S5B). The induction of the potassium ion transporters at P16 reflects the establishment of the unique mechanosensitive transducer channels at the tip of hair cell stereocilia that allow K+ ions to flow into cells. This is a key initial step in the cascade of sound processing. Another cluster of 1,409 induced genes whose expression is markedly higher in the SOC is enriched for regulators of synaptic transmission, levels of neurotransmitters, and calcium ion homoeostasis (Figure 4; Table 2; Table S5B). Such induction of genes implicated in calcium ion homoeostasis during development has previously been reported for the calcium-binding proteins parvalbumin and calbindin in the rat SOC [34,35]. A smaller cluster of 225 that showed full induction in the SOC already at P0 was enriched for genes involved in neuropeptide signalling pathways. As for the induced transcriptional program that is shared by the cochlea and the SOC, a cluster of 560 genes that were similarly activated in both organs was enriched for axon ensheathment/myelination genes.

**Table 2.** GO enrichment for clusters of induced DEGs.

Cluster	GO category	EF	pval	pval.adj
Higher in SOC	regulation of synaptic transmission	2.84	5.46E–18	1.25E–14
	neurotransmitter transport	2.92	1.98E–12	1.82E–09
	calcium ion homoeostasis	2.1	3.78E–08	8.26E–06
Higher in SOC (earlier)	neuropeptide signalling pathway	10.4	4.63E–06	1.96E–03
Higher in cochlea (earlier)	sensory perception of sound	4.9	7.16E–15	2.80E–11
Higher in cochlea (later)	potassium ion transport	3.89	2.34E–07	3.92E–04
	T cell mediated cytotoxicity	10.15	3.92E–06	1.88E–03
Similar	ensheathment of neurons	4.74	6.80E–09	1.22E–05

**Figure 5.**
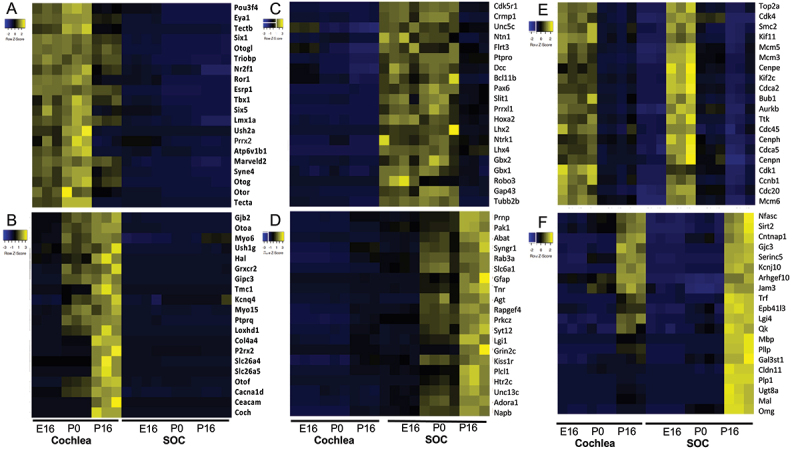
Key regulator and effector genes that are repressed and induced in the organ-specific and shared transcriptional programs in the developing auditory system. Key cochlea-specific repressed (a) or induced (b) genes. Key SOC-specific repressed (c) or induced (d) genes. Key genes that are repressed (E) or induced (F) during maturation of both the cochlea and the SOC.

Taken together, our transcriptomic analysis identifies the main transcriptional programs and biological processes that are induced and repressed during cochlea and SOC development and highlights key regulators and effector genes that carry out these processes (Figure 5). Notably, two clusters detected by our analysis were markedly enriched for deafness genes. First, the cluster of shared repressed DEGs with higher absolute expression levels in the cochlea contained 37 deafness genes (enrichment factor (EF) for deafness genes = 3.7; p-value = 4.09E–12 using hypergeometric tail; Fig. 5a). Second, the cluster of shared induced DEGs with higher absolute expression levels in the cochlea contained 35 deafness genes (EF for deafness genes = 3.77; p-value = 1.06E–11; Figure 5b). Thus, this analysis delineates two distinct pivotal molecular modules of deafness genes: one module comprises deafness genes whose expression level peaks at P0 and are later repressed in mature cochlea (P16). These genes include *Pou3f4*, *Eya1*, *Six1*, *Six5*, *Tbx1*, *Lmx1a*, *Nrf2f1*, *Esrp1* and *Prrx2* that function as transcription factors and regulators; *Triobp*, *Otogl*, *Otog*, *Ush2a*, *Syne4*, *Marveld2*, *Otor* and *Tecta* that encode for structural proteins; and *Atp6v1b1* and *Ror1* that are associated with neuronal function (Figure 5a). The second module comprises deafness genes whose expression level peaks in the mature cochlea (P16), and they encode proteins with very different roles than the deafness genes of the first module: the late-induced deafness genes carry out basic functions of the mature/fully-functional cochlea, mostly categorized with structural or neuronal function, including *Gjb2*, *Oto2*, *Myo5*, *Ush1g*, *Grxcr2*, *Tmc1*, *Myo15*, *Ptprq, Loxhd1, Col4a4, Cacna1d*, and *Coch* or neuronal associated, including *Kcnq4, P2rx2, Slc26a4, Otof*, and *Cacna1d*. Other genes in this group are the regulatory *Gipc3*, the motor protein *Slc26a5*, and *Hal*, an enzyme associated with catabolism (Figure 5b). As observed for SOC-specific developmental programs, the module that is shut-off at P16, predominantly includes key genes for development of neuronal circuits, axonal pathfinding, and neuronal cell type specification, including *Crmp1, Unc5, Ntn1, DCC, Robo3, Slit1, Prrxl1, Flrt3, Lhx2, Lhx4*, *Pax6, Hoxa2, Gb1*, and *Gbx2* (Figure 5c). The program induced in the mature SOC includes key regulators and effectors of synaptic transmission such as *Abat, Syngr1, Rab3a, Slc6a1*, *Syt12, Lgi1, Grin2c, Htr2c, Unc13c*, and *Napb* (Figure 5d). Like the transcriptional module genes with similar absolute expression levels in the two organs, the repressed module contains major genes of all cell-cycle phases, including *Cdk1* and *Mcm3/5* (S phase), *Cdk4* and *Ccnb1* (G2/M), and *Cdc20, Cdca2, Bub1* and *Kif2c/11* (M phase) (Figure 5e). The module induced equally in both the cochlea and the SOC contains the myelination genes *Serinc5*, *Epb41l3, Mal, Omg, Gjc3*, and *Arhgef10* (Figure 5f).

### Regulation between miRNAs and target genes in SE and SOC

**Figure 6.**
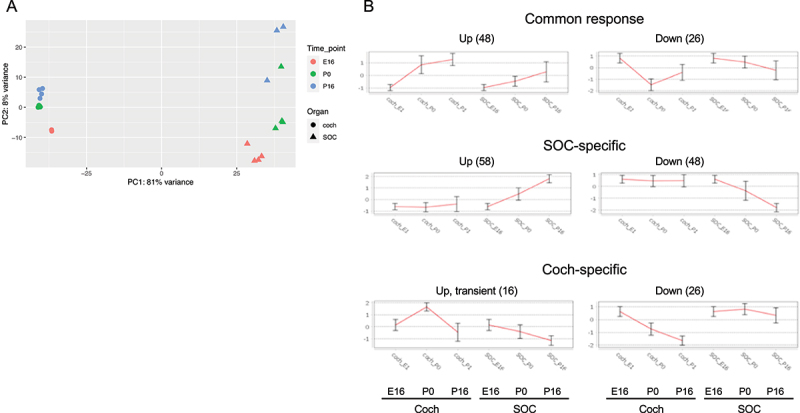
miRNA expression profiles in the developing SOC and cochlea. (a) PCA of the miR-seq data shows that the main component (PC1) is associated with organ-specific expression programs, while the second component (PC2) reflects developmental programs that are shared by the cochlea and the SOC. (b) The main clusters detected for the differentially expressed miRnas in our dataset.

Our next goal was to explore the modulation of microRNA (miR) expression during the development of the cochlea and SOC. To this end, we conducted miR-seq on cochlea and SOC samples from the same developmental time points as in our RNA-seq experiment (E16, P0, and P16). Again, the experiments assessed 3–4 independent samples. Overall, the results detected the expression of 319 miRNAs (Table S6). As before, PCA detected a clear separation of samples by organ, with a secondary division by developmental phase (Figure 6a). Overall, miRNA expression profiles showed greater distinction between the two organs than the RNA-seq gene expression profiles (PC1 in miRNA-seq accounts for 81% of expression variation (Figure 6a) compared to 64% in the RNA-seq data (Figure 2)). Differential expression analysis collectively identified 223 DE miRNAs (Table S7) which could be clustered into organ-specific or shared programs of miRNA modulation (Figure 6b).

**Figure 7.**
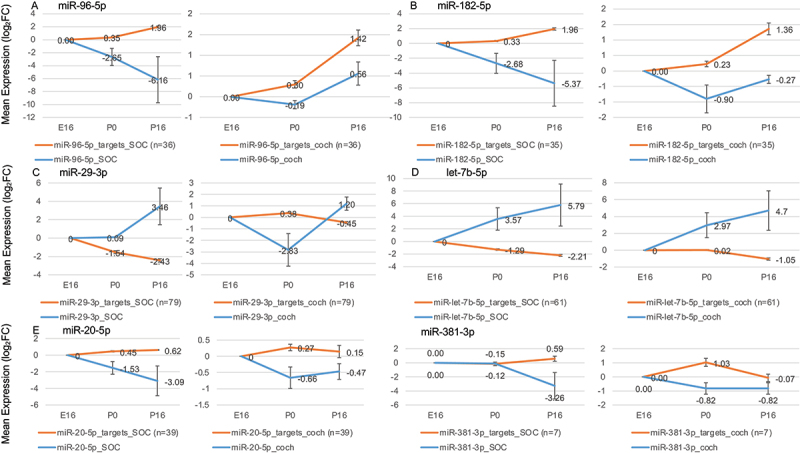
miRs with putative regulatory roles in the development of the auditory system. Each panel shows the mean expression of the miR in the cochlea and the SOC, as well as the mean expression of its predicted target genes (which were assigned to the cluster where the miRNA targets are over-represented). Error bars show ± SD.

As the next stage, we screened the expression programs for miRNA and protein-coding genes in the developing cochlea and SOC for possible regulatory interactions. To this end, we first examined the protein-coding gene clusters (Figures 3–4) for enrichment of predicted targets of specific miRNAs. Since miRNAs often destabilize their target transcripts, the expression of miRNAs that play a regulatory role in the development of the cochlea and the SOC might be expected to exhibit a negative correlation with the expression pattern of the target genes. We therefore examined the expression profile of each miRNA with enrichment of the predicted targets in a certain gene cluster for any significant negative correlation. Overall, this analysis detected six miRNAs, miR-20, miR-29, miR-96, miR-182, miR-381, and let-7b (p < 0.01 for the enrichment of predicted targets and *r* < −0.4 for the correlation between the mean expression patterns of the miRNA and the predicted targets, Table S8). Interestingly, these include miR-96, whose germline mutations cause deafness in humans and mice [17,18]. The expression of miR-96 was markedly repressed during the maturation of the SOC, while the expression of its predicted targets was significantly induced, suggesting their de-repression (Figure 7a). Reassuringly, miR-182, which is part of the miR-96 gene cluster, and thus is co-expressed, showed a similar expression pattern (Figure 7b). The genes *Gria3, Kcna1 Dock9, Ank3*, and *Syp*, which are regulated by miR-96 and miR-182, are mainly involved in neurotransmission. miR-183, which is also a member of the miR-96 cluster, showed the same trend, but did not reach our statistical cut-off for differential expression. This is in agreement with different half-lives of miRNAs from the same cluster [36]. Interestingly, let-7b and miR-29-3p displayed the opposite trend in that their expression was significantly induced during cochlea and SOC maturation, while the expression of their predicted target genes was down-regulated (Figure 7c-d). Notably, the target genes of these miRNAs are involved in regulation of neuronal circuit formation (*Plxnd1, Unk, Ephb3, Sema3f, Lmx1a, Lrig3, Tenm3, Chsy1, Sdk1, Mafb, Glis2*), cell survival (*Ybx3, Emp1, Tnfrsf1a, Glis2*), and ubiquitination (*Dtx2, Usp21, Fbxl12, Ubtd2, Usp42*) (Table S9). In addition, let-7b targets *Tmem2*, which is implicated in human deafness [37].

## Discussion

A comprehensive understanding of the genetic factors governing the development of the hearing system is essential for the development of therapeutic approaches for hearing loss. While hundreds of genes are known to cause hearing loss, many cases of genetic deafness remain unresolved or with an unknown aetiology [5,6]. Auditory prostheses, such as cochlear implants, are designed to bypass the peripheral hearing system, and have varying effectiveness in restoring hearing for individual patients [38,39]. Previous transcriptome studies have generally focused either on the central or the peripheral hearing system [23,40–43], but there has been little attention given to the genetic elements that are common to these two systems. As an exception, one of our previous studies, which focused on 12 cochlea-abundant miRNAs, revealed widespread expression of these miRNAs in the auditory brainstem [24].

Both the inner ear and the SOC arise from the ectoderm during embryogenesis. Inner ear formation begins with the appearance of the otic placode, which in turn evolves into the otic vesicle. This then gives rise to the cochlea and vestibule, including the SE, and the spiral ganglion [44,45]. During brain development, the nuclei of the SOC develop from the rhombencephalon, an embryonic transient structure that is subdivided along the anterior-posterior axis into segmental compartments, named rhombomeres (r) [46]. Interestingly, the SOC derives mainly from r5 with a minor contribution of r4 and the otic placode develops in close spatial proximity to the rhombencephalon adjacent to r5/6. The observation that signalling from the rhombencephalon is essential for induction and development of the otic placode and the enriched of peripheral deafness genes in the SOC suggests a molecular cross talk and a shared gene regulatory network in sensory and neuronal cells of the auditory system [15,47,48]. In agreement, transcription factors such as Atoh1 [49], Lmx1a/b [50], Neurog1 and Neurod1 [51] or Isl1 [52] are essential for both the peripheral and central auditory systems.

In this study, we correlated the expression pattern of both mRNAs and miRNAs from the SE and the SOC at three significant developmental time points. We began by using RNA-seq data to identify genes that are differentially expressed (DEGs) during development and divide them into clusters. Such DEGs are likely to play a role in the proper development of the auditory system. Interestingly, the profiles of most of the DEGs were common to the SE and SOC, with only relatively small groups displaying organ-specific expression. This observation confirms the close developmental connection between the two organs. GO enrichment annotations of each cluster to characterize the main processes, revealed that the group of genes down regulated during the development of both organs is enriched for genes that function in all cell-cycle phases. This reflects the loss of the ability to proliferate during terminal differentiation and organ maturation. In contrast, down regulated genes that are highly expressed in the cochlea relative to the SOC area enriched for genes with functions in early inner-ear development, mesenchyme development, morphogenesis of epithelium, and the Wnt receptor-signalling pathway. In this context, Wnt-signalling is essential for inner ear development, because it is important for dorsal-ventral patterning of the otic vesicle [53]. Down regulated genes preferably expressed in the SOC displayed a prominence of GO expressions representing key roles in axonogenesis, axon guidance, and the neuropeptide signalling pathway. These include Robo3, which has been shown to be important for axonal midline crossing in the SOC [54].

The induced DEGs exhibit five main profiles. The upregulated groups with higher expression in the cochlea are dominated by genes that function in the sensory perception of sound, potassium ion transport, and the adaptive immune response, where potassium transport is a key step towards creating action potentials in the cochlea to transduce sound [55]. Similarly, although the adaptive immune response in the SE remains unclear, it holds great importance for SE repair after trauma [56]. On the other hand, induced genes more prominent in the SOC presented enrichment for regulators of synaptic transmission, neurotransmitter levels and calcium ion homoeostasis. Calcium signalling was previously shown to play an essential role for proper development of the SOC [57,58]. On the other hand, groups induced genes that favour expression in the SOC prove to be enriched for regulators of synaptic transmission, neurotransmitter levels, and calcium ion homoeostasis. Expectedly, the upregulated group with a similar expression in the two organs is enriched for axon ensheathment, as this process is a hallmark of functional maturation of neuronal circuits [59].

Both the induced and repressed groups of DEGs with higher expression in the cochlea were enriched for deafness genes. This cochlear bias can be explained in several ways. First, it could suggest that cochlear development is more susceptible to changes in genes expression. Alternatively, it could imply that some central hearing loss pathologies evolve at a different age than that generally examined, or that they are not as common. A third option is that the SOC is much more complex than the SE with respect to cell types, with the lateral superior olive (LSO) alone comprising seven different cell types [60]. It is therefore possible that the complex mRNA pattern of the SOC may ‘dilute’ deafness genes if they are expressed by a specific cell type]. Finally, it might be difficult to detect dysfunctions that are limited or restricted to the central auditory system, making central deafness genes harder to identify and study. Furthermore, the channelling of auditory information into various convergent and divergent subcircuits may render the central auditory system more resilient than the cochlea [52]. Our analysis revealed these deafness genes either as a peak at P0 with later repression at P16 or as a peak at P16; that is, they are active either as the SE cells complete differentiation or at hearing onset. Combining these expression patterns has the potential to detect a whole variety of new deafness genes.

With regard to the developmental expression patterns of miRNAs in the SE and SOC, PCA analysis of miRNA-seq data revealed strong organ and tissue-specific miRNA expression [61]. These could be clustered into common and organ specific groups, and screened for miRs with predicted target enrichment in DEG clusters. Correlating the increase of miRNAs with a downregulation of the target gene (or vice versa) allowed us to identify six miRNAs (miR-20, miR-29, miR-96, miR-182, miR 381, and let-7b) that are likely to regulate protein coding genes during SE and SOC development.

Among them is miR-96, a miRNA with well-established functions in both the SE and SOC. This renders the other five miRNAs also promising candidates for critical functions in both organs. Exemplarily, miR-20a regulates the expression of the Cyclin D protein and participates in the RGMa-RhoA pathway [62,63]. Cyclin D overexpression in the mature cochlea leads to the production of supernumerary cells in the inner ear SE [64], while under expression of RhoA causes apical abnormalities in outer hair cells (OHCs) [65]. A second miRNA identified as of interest, let-7b, is part of the -let-7 family. Members of the let-7 family have been described as potential regulators of de-differentiation in the inner ear hair cell regeneration in the newt [66]. In addition, overexpression of let-7 during chicken basilar papilla development postponed Atoh1 induction and reduced pro-sensory cell proliferation [67].

RNA-seq data provide unbiased information, thereby enabling comparisons and quantification. This approach can dissect out regulatory circuits that might not be visible in other ways. Here, we used tissues derived from mice to examine changes between different developmental time points, in two different parts of the hearing pathway. The mouse is a well-established model for hearing research [68] and has been used extensively to discover new deafness genes and explore possible genetic therapy [69]. While using a mouse model allows broad comparisons and access to the required tissues, the inherent limitation is that a model animal cannot fully recapitulate the conditions of human hearing [70]. Nevertheless, the microRNAs we detected, as well as their putative target genes predicted by TargetScan, are conserved between human and mouse.

One limitation of our study is that it is based on the analysis of bulk SOC and cochlear SE tissues. In principle, observed changes in expression levels in bulk transcriptomic analyses can stem from either cell-intrinsic modulation of gene expression programs or from changes in cellular composition of the analysed tissues. Follow-up, single-cell microRNA analysis of the cochlea SE and the SOC should shed light on which of the changes we observed in the expression levels of microRNAs and target genes result from which mechanism.

In conclusion, we present here an integrated RNA-seq and miRNA-seq dataset obtained from organs of the peripheral and central hearing systems. This is accompanied by a comprehensive analysis of the common and organ specific developmental gene expression profiles at three critical developmental time points. Furthermore, our analysis identified several miRNAs that show similar patterns of expression in both organs, together with an inverse correlation to significant hearing loss genes predicted as their targets. As exemplified by the identification of miR-96, the common pathways highlighted by this study provide valuable insights into the complementary development of the peripheral and central hearing systems.

## Supplementary Material

Supplemental Material

## Data Availability

GEO accession number GSE221547

## References

[1] Morton CC, Nance WE. Newborn hearing screening — a silent revolution. N Engl J Med. 2006;354(20):2151–2164. doi: 10.1056/NEJMra050700 16707752

[2] Quaranta N, Coppola F, Casulli M, et al. The prevalence of peripheral and central hearing impairment and its relation to cognition in older adults. Audiol Neurootol. 2014;19 Suppl 1:10–14. 25733360 10.1159/000371597

[3] Haile LM, Kamenov K, Briant PS, et al. Hearing loss prevalence and years lived with disability, 1990–2019: findings from the Global Burden of disease study 2019. The Lancet. 2021;397(10278):996–1009. doi: 10.1016/S0140-6736(21)00516-X PMC7960691 33714390

[4] Nance WE. The genetics of deafness. Ment Retard Dev Disabil Res Rev. 2003;9(2):109–119. doi: 10.1002/mrdd.10067 12784229

[5] Abu Rayyan A, Kamal L, Casadei S, et al. Genomic analysis of inherited hearing loss in the Palestinian population. Proc Natl Acad Sci, USA. 2020;117(33):20070–20076. doi: 10.1073/pnas.2009628117 32747562 PMC7443947

[6] Brownstein Z, Gulsuner S, Walsh T, et al. Spectrum of genes for inherited hearing loss in the Israeli Jewish population, including the novel human deafness gene *ATOH1*. Clin Genet. 2020;98(4):353–364. doi: 10.1111/cge.13817 33111345 PMC8045518

[7] Shearer AE, Shen J, Amr S, et al. A proposal for comprehensive newborn hearing screening to improve identification of deaf and hard-of-hearing children. Genet Med. 2019;21(11):2614–2630. doi: 10.1038/s41436-019-0563-5 31171844 PMC6831511

[8] Pickles JO. Auditory pathways: anatomy and physiology. Handb Clin Neurol. 2015;129:3–25. 25726260 10.1016/B978-0-444-62630-1.00001-9

[9] Grothe B, Pecka M, McAlpine D. Mechanisms of sound localization in mammals. Physiol Rev. 2010;90(3):983–1012. doi: 10.1152/physrev.00026.2009 20664077

[10] Kopp-Scheinpflug C, Sinclair JL, Linden JF. When sound stops: offset responses in the auditory system. Trends Neurosci. 2018;41(10):712–728. doi: 10.1016/j.tins.2018.08.009 30274606

[11] Frank MM, Goodrich LV. Talking back: development of the olivocochlear efferent system. Wiley Interdiscip Rev Dev Biol. 2018;7(6):e324. doi: 10.1002/wdev.324 29944783 PMC6185769

[12] Willaredt MA, Schluter T, Nothwang HG. The gene regulatory networks underlying formation of the auditory hindbrain. Cell Mol Life Sci. 2015;72(3):519–535. doi: 10.1007/s00018-014-1759-0 25332098 PMC11113740

[13] Whitfield TT. Development of the inner ear. Curr Opin Genet Dev. 2015;32:112–118. doi: 10.1016/j.gde.2015.02.006 25796080

[14] Zine A, Fritzsch B. Early steps towards hearing: placodes and sensory development. Int J Mol Sci. 2023;24(8):24. doi: 10.3390/ijms24086994 PMC10139157 37108158

[15] Ehmann H, Hartwich H, Salzig C, et al. Time-dependent gene expression analysis of the developing superior olivary complex. J Biol Chem. 2013;288(36):25865–25879. doi: 10.1074/jbc.M113.490508 23893414 PMC3764792

[16] Michalski N, Petit C. Genes involved in the development and physiology of both the peripheral and central auditory systems. Annu Rev Neurosci. 2019;42(1):67–86. doi: 10.1146/annurev-neuro-070918-050428 30699050

[17] Lewis MA, Quint E, Glazier AM, et al. An ENU-induced mutation of miR-96 associated with progressive hearing loss in mice. Nat Genet. 2009;41(5):614–618. doi: 10.1038/ng.369 19363478 PMC2705913

[18] Mencia A, Modamio-Hoybjor S, Redshaw N, et al. Mutations in the seed region of human miR-96 are responsible for nonsyndromic progressive hearing loss. Nat Genet. 2009;41(5):609–613. doi: 10.1038/ng.355 19363479

[19] Schluter T, Berger C, Rosengauer E, et al. miR-96 is required for normal development of the auditory hindbrain. Hum Mol Genet. 2018;27(5):860–874. doi: 10.1093/hmg/ddy007 29325119

[20] Zolboot N, Du JX, Zampa F, et al. MicroRNAs instruct and maintain cell type diversity in the nervous system. Front Mol Neurosci. 2021;14:646072. doi:10.3389/fnmol.2021.646072 33994943 PMC8116551

[21] Friedman LM, Dror AA, Mor E, et al. MicroRNAs are essential for development and function of inner ear hair cells in vertebrates. Proc Natl Acad Sci, USA. 2009;106(19):7915–7920. doi: 10.1073/pnas.0812446106 19416898 PMC2683084

[22] Rosengauer E, Hartwich H, Hartmann AM, et al. Egr2: cre mediated conditional ablation of dicer disrupts histogenesis of mammalian central auditory nuclei. PLoS One. 2012;7(11):e49503. doi: 10.1371/journal.pone.0049503 23152916 PMC3495878

[23] Rudnicki A, Isakov O, Ushakov K, et al. Next-generation sequencing of small RNAs from inner ear sensory epithelium identifies microRnas and defines regulatory pathways. BMC Genomics. 2014;15(1):484. doi: 10.1186/1471-2164-15-484 24942165 PMC4073505

[24] Krohs C, Bordeynik-Cohen M, Messika-Gold N, et al. Expression pattern of cochlear microRnas in the mammalian auditory hindbrain. Cell Tissue Res. 2021;383(2):655–666. doi: 10.1007/s00441-020-03290-x 33156384 PMC7904729

[25] Dobin A, Davis CA, Schlesinger F, et al. STAR: ultrafast universal RNA-seq aligner. Bioinformatics. 2013;29(1):15–21. doi: 10.1093/bioinformatics/bts635 23104886 PMC3530905

[26] Love MI, Huber W, Anders S. Moderated estimation of fold change and dispersion for RNA-seq data with DESeq2. Genome Biol. 2014;15(12):550. doi: 10.1186/s13059-014-0550-8 25516281 PMC4302049

[27] Hait TA, Maron-Katz A, Sagir D, et al. The EXPANDER integrated platform for transcriptome analysis. J Mol Biol. 2019;431(13):2398–2406. doi: 10.1016/j.jmb.2019.05.013 31100387

[28] Wu T, Hu E, Xu S, et al. clusterProfiler 4.0:a universal enrichment tool for interpreting omics data. Innovation (Camb). 2021;2(3):100141. doi: 10.1016/j.xinn.2021.100141 34557778 PMC8454663

[29] Babicki S, Arndt D, Marcu A, et al. Heatmapper: web-enabled heat mapping for all. Nucleic Acids Res. 2016;44(W1):W147–53. doi: 10.1093/nar/gkw419 27190236 PMC4987948

[30] Langmead B, Trapnell C, Pop M, et al. Ultrafast and memory-efficient alignment of short DNA sequences to the human genome. Genome Biol. 2009;10(3):R25. doi: 10.1186/gb-2009-10-3-r25 19261174 PMC2690996

[31] Fromm B, Hoye E, Domanska D, et al. MirGeneDB 2.1: toward a complete sampling of all major animal phyla. Nucleic Acids Res. 2022;50(D1):D204–D10. doi: 10.1093/nar/gkab1101 34850127 PMC8728216

[32] Agarwal V, Bell GW, Nam JW, et al. Predicting effective microRNA target sites in mammalian mRnas. Elife. 2015;4:4. doi:10.7554/eLife.05005 PMC4532895 26267216

[33] Groves AK, Fekete DM. Shaping sound in space: the regulation of inner ear patterning. Development. 2012;139(2):245–257. doi: 10.1242/dev.067074 22186725 PMC3243092

[34] Friauf E. Transient appearance of calbindin-D28k-positive neurons in the superior olivary complex of developing rats. J Comp Neurol. 1993;334(1):59–74. doi: 10.1002/cne.903340105 8408759

[35] Lohmann C, Friauf E. Distribution of the calcium-binding proteins parvalbumin and calretinin in the auditory brainstem of adult and developing rats. J Comp Neurol. 1996;367(1):90–109. doi: 10.1002/(SICI)1096-9861(19960325)367:1<90:AID-CNE7>3.0.CO;2-E 8867285

[36] Zhou L, Lim MYT, Kaur P, et al. Importance of miRNA stability and alternative primary miRNA isoforms in gene regulation during Drosophila development. Elife. 2018;7: doi: 10.7554/eLife.38389 PMC6066331 30024380

[37] Scott DA, Drury S, Sundstrom RA, et al. Refining the DFNB7-DFNB11 deafness locus using intragenic polymorphisms in a novel gene, TMEM2. Gene. 2000;246(1–2):265–274. doi: 10.1016/s0378-1119(00)00090-1 10767548

[38] Peixoto MC, Spratley J, Oliveira G, et al. Effectiveness of cochlear implants in children: long term results. Int J Pediatr Otorhinolaryngol. 2013;77:462–468. doi:10.1016/j.ijporl.2012.12.005 23291164

[39] Sharma SD, Cushing SL, Papsin BC, et al. Hearing and speech benefits of cochlear implantation in children: a review of the literature. Int J Pediatr Otorhinolaryngol. 2020;133:109984. 32203759 10.1016/j.ijporl.2020.109984

[40] Chadly DM, Best J, Ran C, et al. Developmental profiling of microRnas in the human embryonic inner ear. PLoS One. 2018;13(1):e0191452. doi: 10.1371/journal.pone.0191452 29373586 PMC5786302

[41] Kolla L, Kelly MC, Mann ZF, et al. Characterization of the development of the mouse cochlear epithelium at the single cell level. Nat Commun. 2020;11(1):2389. doi: 10.1038/s41467-020-16113-y 32404924 PMC7221106

[42] Saleh AJ, Nothwang HG. Differential expression of microRnas in the developing avian auditory hindbrain. J Comp Neurol. 2021;529(15):3477–3496. doi: 10.1002/cne.25205 34180540

[43] Petitpre C, Faure L, Uhl P, et al. Single-cell RNA-sequencing analysis of the developing mouse inner ear identifies molecular logic of auditory neuron diversification. Nat Commun. 2022;13(1):3878. doi: 10.1038/s41467-022-31580-1 35790771 PMC9256748

[44] Freyer L, Aggarwal V, Morrow BE. Dual embryonic origin of the mammalian otic vesicle forming the inner ear. Development. 2011;138(24):5403–5414. doi: 10.1242/dev.069849 22110056 PMC3222214

[45] Mao Y, Reiprich S, Wegner M, et al. Targeted deletion of Sox10 by Wnt1-cre defects neuronal migration and projection in the mouse inner ear. PLoS One. 2014;9(4):e94580. doi: 10.1371/journal.pone.0094580 24718611 PMC3981815

[46] Nothwang HG. Evolution of mammalian sound localization circuits: a developmental perspective. Prog Neurobiol. 2016;141:1–24. doi: 10.1016/j.pneurobio.2016.02.003 27032475

[47] Willaredt MA, Ebbers L, Nothwang HG. Central auditory function of deafness genes. Hear Res. 2014;312:9–20. doi: 10.1016/j.heares.2014.02.004 24566090

[48] Elliott KL, Fritzsch B, Yamoah EN, et al. Age-related hearing loss: sensory and neural etiology and their interdependence. Front Aging Neurosci. 2022;14:814528. doi:10.3389/fnagi.2022.814528 35250542 PMC8891613

[49] Maricich SM, Xia A, Mathes EL, et al. Atoh1-lineal neurons are required for hearing and for the survival of neurons in the spiral ganglion and brainstem accessory auditory nuclei. J Neurosci. 2009;29(36):11123–11133. doi: 10.1523/JNEUROSCI.2232-09.2009 19741118 PMC2743121

[50] Chizhikov VV, Iskusnykh IY, Fattakhov N, et al. Lmx1a and Lmx1b are redundantly required for the development of multiple components of the mammalian auditory system. Neuroscience. 2021;452:247–264. 33246067 10.1016/j.neuroscience.2020.11.013 PMC7780644

[51] Elliott KL, Pavlinkova G, Chizhikov VV, et al. Neurog1, Neurod1, and Atoh1 are essential for spiral ganglia, cochlear nuclei, and cochlear hair cell development. Fac Rev. 2021;10:47. 34131657 10.12703/r/10-47 PMC8170689

[52] Filova I, Pysanenko K, Tavakoli M, et al. ISL1 is necessary for auditory neuron development and contributes toward tonotopic organization. Proc Natl Acad Sci, USA. 2022;119(37):e2207433119. doi: 10.1073/pnas.2207433119 36074819 PMC9478650

[53] Munnamalai V, Fekete DM. Wnt signaling during cochlear development. Semin Cell Dev Biol. 2013;24(5):480–489. doi: 10.1016/j.semcdb.2013.03.008 23548730 PMC3690158

[54] Michalski N, Babai N, Renier N, et al. Robo3-driven axon midline crossing conditions functional maturation of a large commissural synapse. Neuron. 2013;78(5):855–868. doi: 10.1016/j.neuron.2013.04.006 23664551

[55] Szeto IYY, Chu DKH, Chen P, et al. SOX9 and SOX10 control fluid homeostasis in the inner ear for hearing through independent and cooperative mechanisms. Proc Natl Acad Sci, USA. 2022;119(46):e2122121119. doi: 10.1073/pnas.2122121119 36343245 PMC9674217

[56] Keithley EM. Inner ear immunity. Hear Res. 2022;419:108518. doi: 10.1016/j.heares.2022.108518 35584985

[57] Ebbers L, Satheesh SV, Janz K, et al. L-type calcium channel Cav1.2 is required for maintenance of auditory brainstem nuclei. J Biol Chem. 2015;290(39):23692–23710. doi: 10.1074/jbc.M115.672675 26242732 PMC4583033

[58] Satheesh SV, Kunert K, Ruttiger L, et al. Retrocochlear function of the peripheral deafness gene Cacna1d. Hum Mol Genet. 2012;21(17):3896–3909. doi: 10.1093/hmg/dds217 22678062

[59] Stassart RM, Mobius W, Nave KA, et al. The axon-myelin unit in development and degenerative disease. Front Neurosci. 2018;12:467. doi:10.3389/fnins.2018.00467 30050403 PMC6050401

[60] Rietzel HJ, Friauf E. Neuron types in the rat lateral superior olive and developmental changes in the complexity of their dendritic arbors. J Comp Neurol. 1998;390(1):20–40. doi: 10.1002/(SICI)1096-9861(19980105)390:1<20:AID-CNE3>3.0.CO;2-S 9456173

[61] Ludwig N, Leidinger P, Becker K, et al. Distribution of miRNA expression across human tissues. Nucleic Acids Res. 2016;44(8):3865–3877. doi: 10.1093/nar/gkw116 26921406 PMC4856985

[62] Ghosh T, Aprea J, Nardelli J, et al. MicroRNAs establish robustness and adaptability of a critical gene network to regulate progenitor fate decisions during cortical neurogenesis. Cell Rep. 2014;7(6):1779–1788. doi: 10.1016/j.celrep.2014.05.029 24931612

[63] Feng Y, Duan C, Luo Z, et al. Silencing miR-20a-5p inhibits axonal growth and neuronal branching and prevents epileptogenesis through RGMa-RhoA-mediated synaptic plasticity. J Cell Mol Med. 2020;24(18):10573–10588. doi: 10.1111/jcmm.15677 32779334 PMC7521253

[64] Tarang S, Pyakurel U, Weston MD, et al. Spatiotemporally controlled overexpression of cyclin D1 triggers generation of supernumerary cells in the postnatal mouse inner ear. Hear Res. 2020;390:107951. doi:10.1016/j.heares.2020.107951 32244147 PMC7204889

[65] Anttonen T, Belevich I, Laos M, et al. Cytoskeletal stability in the auditory organ in vivo: RhoA is dispensable for wound healing but essential for hair cell development. eNeuro. 2017;4(5):4. doi: 10.1523/ENEURO.0149-17.2017 PMC5602105 28929130

[66] Tsonis PA, Call MK, Grogg MW, et al. MicroRNAs and regeneration: Let-7 members as potential regulators of dedifferentiation in lens and inner ear hair cell regeneration of the adult newt. Biochem Biophys Res Commun. 2007;362(4):940–945. doi: 10.1016/j.bbrc.2007.08.077 17765873 PMC2683343

[67] Evsen L, Li X, Zhang S, et al. *Let-7* miRnas inhibit CHD7 expression and control auditory-sensory progenitor cell behavior in the developing inner ear. Development. 2020;147(15):147. doi: 10.1242/dev.183384 PMC7438007 32816902

[68] Ohlemiller KK, Jones SM, Johnson KR. Application of mouse models to research in hearing and balance. J Assoc Res Otolaryngol. 2016;17(6):493–523. doi: 10.1007/s10162-016-0589-1 27752925 PMC5112220

[69] Taiber S, Gwilliam K, Hertzano R, et al. The genomics of auditory function and disease. Annu Rev Genomics Hum Genet. 2022;23(1):275–299. doi: 10.1146/annurev-genom-121321-094136 35667089 PMC10644960

[70] Tona R, Lopez IA, Fenollar-Ferrer C, et al. Mouse models of human pathogenic variants of TBC1D24 associated with non-syndromic deafness DFNB86 and DFNA65 and syndromes involving deafness. Genes (Basel). 2020;11(10):11. doi: 10.3390/genes11101122 PMC7598720 32987832

